# Patient Recruitment System for Clinical Trials: Mixed Methods Study About Requirements at Ten University Hospitals

**DOI:** 10.2196/28696

**Published:** 2022-04-20

**Authors:** Kai Fitzer, Renate Haeuslschmid, Romina Blasini, Fatma Betül Altun, Christopher Hampf, Sherry Freiesleben, Philipp Macho, Hans-Ulrich Prokosch, Christian Gulden

**Affiliations:** 1 Core Unit Data Integration Center University Medicine Greifswald Greifswald Germany; 2 Institute for Community Medicine University Medicine Greifswald Greifswald Germany; 3 Institute of Medical Biometry and Statistics Faculty of Medicine and Medical Center University of Freiburg Freiburg Germany; 4 Institute of Medical Informatics University of Giessen Giessen Germany; 5 Medical Informatics Group University Hospital Frankfurt Frankfurt Germany; 6 Medical Informatics Institute of Medical Biostatistics, Epidemiology and Informatics University Medical Center of the Johannes Gutenberg-University Mainz Mainz Germany; 7 Medical Informatics Friedrich-Alexander University Erlangen-Nürnberg Erlangen Germany

**Keywords:** patient recruitment system, clinical trial recruitment support system, recruitment, patient screening, requirements, user needs, clinical trial, interview, survey, electronic support, clinical information systems, eHealth

## Abstract

**Background:**

Clinical trials are the gold standard for advancing medical knowledge and improving patient outcomes. For their success, an appropriately sized cohort is required. However, patient recruitment remains one of the most challenging aspects of clinical trials. Information technology (IT) support systems—for instance, patient recruitment systems—may help overcome existing challenges and improve recruitment rates, when customized to the user needs and environment.

**Objective:**

The goal of our study is to describe the status quo of patient recruitment processes and to identify user requirements for the development of a patient recruitment system.

**Methods:**

We conducted a web-based survey with 56 participants as well as semistructured interviews with 33 participants from 10 German university hospitals.

**Results:**

We here report the recruitment procedures and challenges of 10 university hospitals. The recruitment process was influenced by diverse factors such as the ward, use of software, and the study inclusion criteria. Overall, clinical staff seemed more involved in patient identification, while the research staff focused on screening tasks. Ad hoc and planned screenings were common. Identifying eligible patients was still associated with significant manual efforts. The recruitment staff used Microsoft Office suite because tailored software were not available. To implement such software, data from disparate sources will need to be made available. We discussed concrete technical challenges concerning patient recruitment systems, including requirements for features, data, infrastructure, and workflow integration, and we contributed to the support of developing a successful system.

**Conclusions:**

Identifying eligible patients is still associated with significant manual efforts. To fully make use of the high potential of IT in patient recruitment, many technical and process challenges have to be solved first. We contribute and discuss concrete technical challenges for patient recruitment systems, including requirements for features, data, infrastructure, and workflow integration.

## Introduction

Medical research requires the involvement of sufficiently sized and eligible patient cohorts. A shortage of participants may result in delays, reduced statistical validity, increased costs, or even the failure of costly trials [1]. Indeed, poor recruitment has been found to be the main reason for trial discontinuation [2,3]. Only 31% of the analyzed clinical trials were able to reach the targeted participant count within the time frame [4,5]. Williams et al [6] analyzed ended trials published on ClinicalTrials.gov and concluded that 57% of those were terminated owing to an insufficient rate of accrual. Those numbers point to a strong need for support, and software tools are a promising approach that may improve effectiveness and efficiency [7-12]. As noted by Beresniak et al [7], optimizing processes and using efficient IT support could reduce costs and allow for more clinical trials to be successfully completed with fewer resources. Software can support the recruitment process in various ways. In this work, we are particularly interested in tools that support the recruitment staff in identifying eligible patients by screening large amounts of routine data, including screening electronic health records (EHRs) for specific criteria. Those systems are commonly called patient recruitment systems (PRS), clinical trial recruitment support systems (CTRSS), or sometimes also called clinical decision–support systems [13].

There are numerous studies on approaches, prototypes, and tools to support patient recruitment. Cuggia et al [12] compared 28 PRS regarding their contributions, limitations, features, and efficacy. Köpcke et al [14] reviewed 79 PRS regarding their design and theorized on the approaches; for example, why they think specific design decisions were taken. A very recent review of Pung and Rienhoff [1] included 36 articles that evaluated recruitment-related electronic systems or described related workflows. A major caveat of these works is that neither their efficiency nor their design have been sufficiently evaluated in a real-world, clinical environment. These publications note improvements in recruitment in terms of effectiveness and efficiency; for example, increased recruitment numbers and time savings. However, only a few systems have been subjected to meaningful evaluation to date.

In their review, Köpcke et al [14] concluded that the utility of a PRS depends on the patient data available and the integration of the PRS into study and clinical workflows, but they also say that this has not been sufficiently explored. Cuggia et al [12] showed that the workflow, organization, and communication as well as users' perception and acceptance have not yet been sufficiently considered. Based on their review of the matter, they identified the physicians' limited time as well as their knowledge and awareness of trials to be some of the major obstacles.

Straube et al [15] identified limited human and technical resources and the high documentation effort as the most prevalent barriers. Schreiweis and Bergh [16] argue that “a detailed analysis of stakeholders’ requirements would help implementing better patient recruitment systems (PRS) in the future” and took a 2-fold approach to do so. They named some functional requirements for PRS, but no information on the context, the methods, or the participant count (N) was provided. Trinczek et al [17] interviewed 6 “domain experts” and identified a set of 23 tasks, with most tasks being related to patient identification. In a later study, Trinczek et al [18] showed that a large proportion of the work is manual and paper-based because the ability to search in the clinical databases is very restricted.

A PRS may be a solution to many of these issues. For an effective and accepted solution, we first need to thoroughly understand the users’ needs, current workflows, tasks, and barriers. Systems that are not well-embedded in the hospital work environment or those that do not answer direct needs are likely to be ineffective or even rejected by the users, which is why involving users in the design process is crucial for the success of these systems [19-21]. As listed above, few studies have evaluated the workflows and tasks, and they all have strong limitations; for example, they included few participants, provide little information about their methods and analysis, or only report on few aspects or requirements. We are not aware of a study that draws a complete picture and has holistically investigated the status quo in patient recruitment, including the recruitment workflows and tasks, as well as the users’ requirements and wishes regarding future PRSs. To extend the existing work, we applied a user-centered research approach and surveyed 56 prospective users and interviewed 33 potential ones—that is, patient recruitment staff—from 10 Medical Informatics in Research and Care in University Medicine (MIRACUM) university hospitals. We performed a qualitative analysis of the state-of-the-art recruitment workflows, procedures, issues, and existing technological support from the 10 sites. Furthermore, we established a collection of user-centric requirements for future patient recruitment systems. We completed this paper with a discussion of technical and functional requirements as well as how and where a PRS may be integrated in the clinical infrastructures and processes.

## Methods

### Methods Overview

We aimed to assess the status quo in patient recruitment to better support it with appropriate IT systems. We predominantly collected quantitative data from a web-based survey and qualitative data based on interviews. Both, the web-based survey and interviews were developed and carried out simultaneously and took place at 10 university hospitals that are part of the MIRACUM consortium.

### Web-based Survey

#### Design

The survey contained different questions about the current, local recruitment workflows, and specifically about the screening tasks and timing as well as the communication of recruitment suggestions. Furthermore, we were interested in different patient recruitment tools: we asked about their attitude toward such systems, the expected usefulness, and specific requirements. The initial set of questions were brainstormed collaboratively within the team. After these questions were transferred to the web-based survey tool, a pretest was conducted allowing all team-members to try the survey and report any issues and feedback. In total, the survey consisted of 16 questions with multiple-choice, rating scale, and free-text answer formats, which were structured thematically on 6 different pages. Each page contained between 3 and 7 items, where all items were mandatory to answer, but contained a “not applicable/can’t say” option. The web-based survey was generated using SoSci Survey and captured data anonymously between December 2018 and June 2019 [22].

#### Participants

To capture as many different workflows and perspectives as possible, we aimed to recruit staff members who (1) were involved in the patient recruitment process and (2) filled different positions across a broad spectrum of wards. The survey was sent out to the members of the MIRACUM consortium, who then redirected it to researchers and clinical staff at their site.

#### Analysis

The survey had a completion rate of 93%. The statistical analysis was anonymously conducted using the R (version 3.6, The R Foundation) [23]. Since 30 questions had to be answered using a rating scale ranging from 1 to 5, we used the sjPlot package to visualize the results [24]. All multiple-choice questions were visualized with simple bar plots using the plotly package [25]. We only analyzed complete answers, and answers pertaining to free-text questions on work experience, job title, age, and work experience were manually preprocessed and grouped into common categories before plotting.

### Interviews

#### Design and Procedure

By means of semistructured interviews, we aimed to gather qualitative insights into the workflows, procedures, tasks and other relevant aspects of patient recruitment. We considered the works of DeMoor [10] and Trinczek [18] when designing 14 questions targeting (1) status quo in recruitment, (2) existing technological support, (3) perceived quality and problems, (4) and requirements for a PRS. On average, the interviews took about 45 minutes.

#### Participants

Overall, we collected data from 33 participants from all 10 hospitals (2-7 interviewees per site). Face-to-face and voice-recorded interviews were conducted with 12 participants who gave written consent. Furthermore, we collected answers in free written form from 21 participants with whom scheduling an in-person interview was not possible such as to reach a larger number of participants.

#### Analysis

Before transcribing the voice-recorded interviews, we anonymized all data. We then applied a content analysis approach, as suggested by Mayring [26].

Two authors then read and independently coded 3 randomly selected interviews into codebooks. In this process, codes were assigned for the respective answers to the questions. If a researcher gave a very similar answer to a question, the same code was used. Afterward, the authors compared the independently created codes and merged the codebooks. Owing to a high coding agreement of 95%, the two authors then proceeded to code the remaining 30 interviews independently (15 each). In the case of incomplete interviews, only the answered questions were considered and also coded, as they contained valuable insights. Unanswered questions were not considered in the evaluation.

### Ethical Approval

This study was ethically approved by the ethics committee of the Friedrich-Alexander-University Erlangen-Nürnberg (approval number 412_18B).

## Results

### Results Overview

Below, we report the results obtained from the interviews and the web-based surveys. We illustrated (1) the procedures currently implemented at the participating hospitals as well as (2) the requirements for future patient recruitment tools. We received 56 complete responses of doctors (n=26, 46%), study coordinators (n=7, 13%), study nurses (n=4, 7%), medical documentalists (n=4, 7%), study assistants (n=2, 4%), scientific and technical staff (n=1 each, 2%), and others (n=4, 7%). Seven participants (13%) did not specify their role. Fourteen respondents were aged 25-34 years, 25 were aged 35-44 years, and 10 were aged 45-54 years. The average number of working experiences in the field of patient recruitment was 12. The interviews were conducted between December 2018 and June 2019. The number of participants by medical specialty and participating site is shown in Table 1.

**Table 1:** Participants by medical specialty and participating site.

Medical specialty or department	Participants, n										
	Dresden	Erlangen	Frankfurt	Freiburg	Gießen	Greifswald	Magdeburg	Mainz	Marburg	Mannheim	Total
Obstetrics and gynecology	2	1	0	0	0	0	0	1	0	0	4
Internal medicine	1	0	1	2	0	1	1	3	0	2	11
Surgery	1	1	0	0	0	0	0	0	0	0	2
Pediatrics	0	1	0	0	1	0	0	0	0	0	2
Urology	0	0	1	0	0	0	1	0	0	1	3
Anesthesiology	0	0	0	0	1	0	0	1	0	0	2
Psychiatry	0	0	0	0	1	0	0	0	0	0	1
Medical genetics	0	0	0	0	1	0	0	0	0	0	1
Neurology	0	0	0	0	0	1	0	0	1	0	2
Radiation oncology	0	0	0	0	0	0	1	0	0	0	1
Ophthalmology	0	0	0	0	0	0	1	1	1	0	3
Coordination Center for Clinical Studies	1	0	0	0	0	1	0	0	1	0	3
(Comprehensive) Cancer Center	1	0	0	0	0	0	0	0	0	0	1
Total	6	3	2	2	4	3	4	6	3	3	

### Current Recruitment Procedures and Infrastructure

#### Communication of Recruiting Trials

Our analysis revealed that the first hurdle was to ensure that all the involved parties were aware of a recruiting trial and its accompanying criteria. This awareness was raised through various channels that were either specific to a department and topic or to certain roles and duties. Our interviewees mentioned that they primarily learned about new trials through regular meetings (n=15), such as the tumor board review, through staff from the same clinic and department (n=9), through staff from other clinics and departments (n=9), through sponsors and industry partners (n=11), through clinical partners (n=2), or through personal networks (n=2). Furthermore, our interviewees mentioned that they learned about new studies in the context of training and courses (n=7) as well as at events of associations, fairs, and congresses (n=4), emails (n=3), telephone or SMS (n=2), or printed mail (n=1). Four interviewees highlighted that whether one knows about and is aware of a trial during everyday work depends on the interest and motivation of the employee.

#### Recruitment Procedures and Difficulties

In this section, we break down and summarize all recruitment procedures.

#### Procedures, Roles, and Tasks

Our interviews revealed that the clinical staff was mainly responsible to look for potential participants and forward suitable patient data for further screening (n=10). Research departments (n=2), coordinators (n=2), and auxiliary personnel (n=2) may also support the search; for example, by going through surgery schedules. When potential participants were identified, the research staff took over the detailed eligibility screening. Clinical investigators (n=8), study nurses (n=6), and assistants (n=3) may also be involved in this step. In contrast, our web-based survey exposed a different trend. According to our respondents, clinicians (n=44, 79%) and clinical investigators (n=45, 80%) were nearly equally involved in the identification of patients. Other staff members (n=31, 55%), study nurses and assistants (n=8, 14%) were also involved in identifying patients. Regarding the screening of patients, results from the interviews and web-based survey were more aligned. The survey showed that this task is assigned primarily to research staff; that is, clinical investigators (n=49, 88%) and study nurses and assistants (n=7, 13%). Furthermore, many of our survey respondents (n=22, 39%) stated that clinicians also take over certain screening duties.

#### Timing

**Figure 1:**
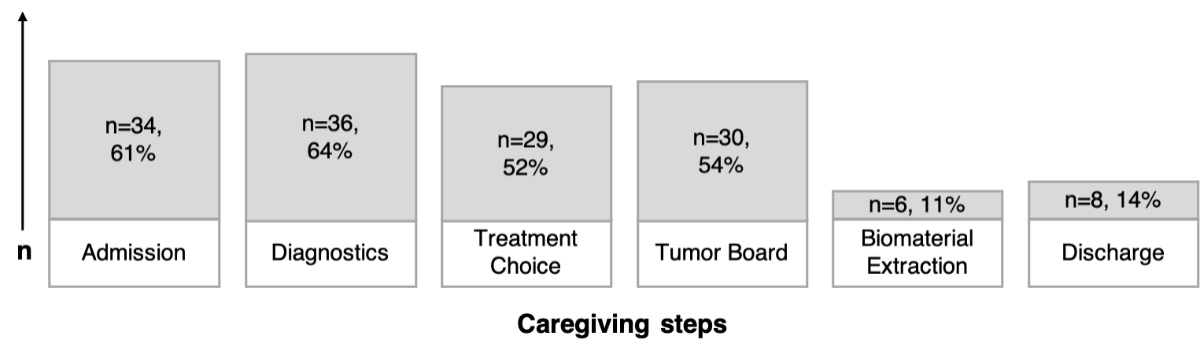
Patients are screened for eligibility at several stages during their hospital stay. The participants could select multiple stages.

In total, 23 interviewees mentioned that they followed a regular, cyclic, or daily recruitment procedure. In an ad-hoc manner, 4 interviewees screened newly moved patients to their ward, and 3 interviewees did not follow regular timing. The results of the web-based survey is presented in Figure 1, which shows the care-giving steps and the survey responses (as percentage values; multiple choices allowed). A patient's suitability for a trial was usually checked during admission, diagnosis, therapy choice, or during the tumor board, and less frequently checked when extracting or analyzing bio-materials, or upon discharge. A number of survey participants stated that they screened patients multiple times, either regularly (n=8, 14%) or without particular timing (n=24, 43%). In total, 19 respondents (34%) stated to screen a patient exactly once and 7 (13%) at every visit.

#### Locations and Sources

**Figure 2:**
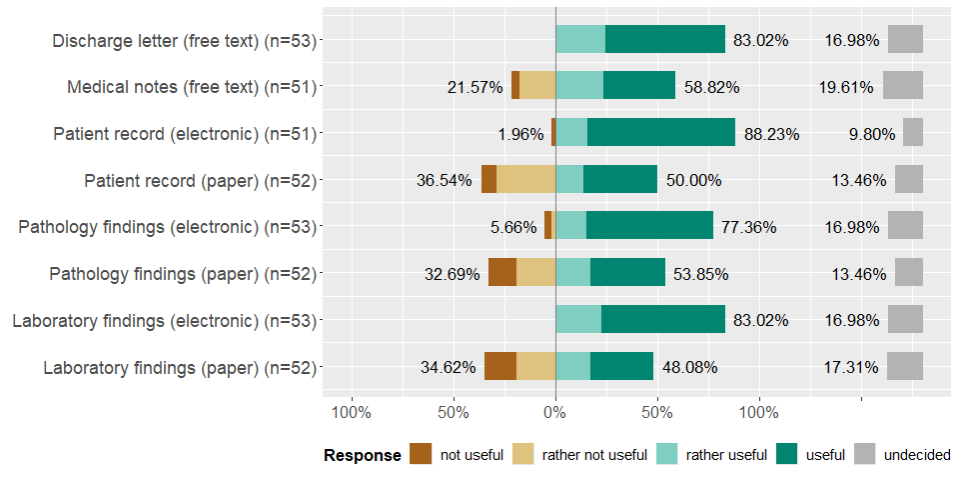
Our participants rated a predefined set of data sources regarding their usefulness for identifying potential participants.

Patients are recruited from their affiliated (n=17) and other departments (n=13), the emergency room (n=15), specific wards (n=9), and through the tumor board (n=12). Patients are recruited through external doctors (n=6) and partners (n=3), advertisements and press (n=5), and referred via other hospitals (n=2). Participants may be searched within (printed) ward-specific lists or schedules (n=4), patient files and medical reports (n=2 each). During the search, they first look at the department or clinic of the patients (n=7) as well as their diagnoses, procedures, laboratory samples and (existing) consents (n=1 each). Then, detailed screening in accordance with the eligibility criteria takes place. In the survey, our respondents rated different sources regarding their suitability for identifying potential participants from little useful (orange) to very useful (cyan). Figure 2 illustrates that electronic patient files, laboratory results, doctor’s letters, and pathological findings are highly useful sources for finding participants. Paper-based or free-text data are regarded as slightly less useful.

#### Difficulties

Overall, our interviewees judged the patient recruitment process as running very well (n=5), well (n=14), bad (n=5), or varying (n=4); for example, depending on the ward and staff. Many interviewees emphasized that sufficient direct communication between employees is essential to the recruitment procedure (n=18). They pointed out that staff-related problems such as staff turnover or shortfalls in motivation, communication, or support from external doctors (n=8) and logistic problems (n=6) are the most prevalent issues in the recruitment process. Furthermore, they mentioned that the data available in the hospital information system (HIS) were insufficient for screening (n=3) or that the eligibility criteria were too specific or complex to search via systems and databases (n=3).

In total, 46% of our interviewees stated that the identification of suitable patients hampers routine care. The screening procedure, consisting of searching for patients to checking all the eligibility criteria, was identified as the most time and labor-intensive step in recruitment (n=16). Informing participant candidates about the trial (n=10) and coordination tasks such as further diagnostics and data retrieval, questionnaires, and appointment management (n=9) were also considered time-consuming.

#### Infrastructure and Systems in Use

In total, 17 interviewees indicated that they already had systems deployed to support the recruitment process, and 13 said that they actually make use of them. Most of those systems were not dedicated to the recruitment process but rather tools developed for other duties and modified to fill the gap. To flag and document the recruited patients, various tools were used: Microsoft Office Tools (n=10), HIS and databases (n=12), SAP (n=7), patient files (n=5), papers (n=5), trial documents (n=5), and the tumor board (n=3). Four participants did not flag recruited patients. Our interviewees emphasized that the systems should provide a good overview (n=3), a good search and query opportunity (n=3), a good overall power (speed, data protection, and user and management function) (n=3). They complained about low data quality and, in particular, that data are insufficiently structured, outdated, and in need of further processing (n=2), which is why the resulting IT tools were not adequately functional.

### Infrastructure Needs and Opportunities

#### Patient Data

Our interviewees mentioned a broad spectrum of patient data that they would like to screen using IT support:

- Diagnoses (n=37): International Classification of Diseases–coded diagnoses (n=20), disease-specific values (n=7; eg, tumor values, heart values, scores, device data, and electrocardiographs), concomitant diseases (n=7), genetic information (n=2), and medical history (n=1)
- Demographic data (n=21): age (n=12), gender (n=6) and ethnicity, marital status, and place of residence (n=1 each)
- Treatment data (n=17): medication (n=6), therapy (n=6), Operation and Procedure Classification System–coded procedures (n=2), the date of surgery (n=2), and clinical findings (n=1)
- Laboratory data (n=12): blood count, heart failure markers, and histology; further laboratory values were indicated but not explicitly named
- Vital signs (n=8): patient's general condition (n=2), height (n=2), weight, implants, organ function, and study participation (n=1 each)

**Figure 3:**
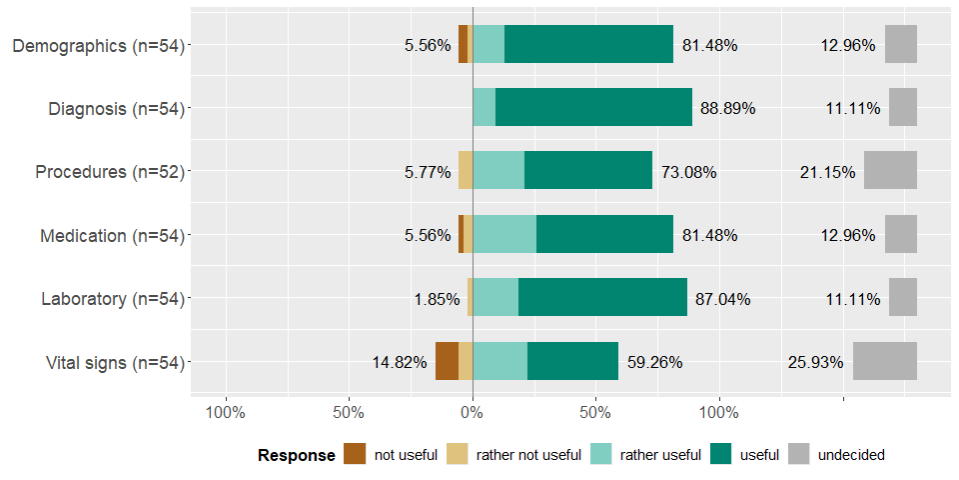
Our participants rated a predefined set of data groups regarding their usefulness for patient identification.

The survey results were overall in line with those of the interviews. Our survey respondents selected the diagnosis as the most important criteria, followed by laboratory data, demographics, medications, and procedures (summarized to treatment above). Vital signs also seemed useful, albeit with a lower priority. Figure 3 shows the respondents' estimated usefulness of the data rated on a 5-item scale.

#### Recruitment Suggestions

For recruitment suggestions, our interviewees wanted to be notified by a system (n=26) or to check suggestions by themselves (n=16). Three interviewees did not want to be notified. Notifications by email (n=9), popups (n=4), highlighting of the patient (n=3), SMS text messages or telephone (n=1), or in any way (n=8) were mentioned. Many interviewees desired a list of all patient suggestions, potentially integrated into the HIS (n=15). The survey results underline these desires: 83% of the respondents wanted a screening list with recruitment proposals and 81% wanted to receive email notifications.

#### Wishes and Requirements

Our interviewees expressed other wishes; that is, they requested functional extensions and optimizations of existing clinical applications (n=18), more sophisticated searches for eligible patients (n=15), and an optimized tracking of included patients (n=3). They especially desired improvements regarding searching, usability, design, interoperability of research and clinical systems (n=9 overall), popups for requesting input of additional research data (n=6), and decision support (n=3).

#### Opinions and Expectations

**Figure 4:**
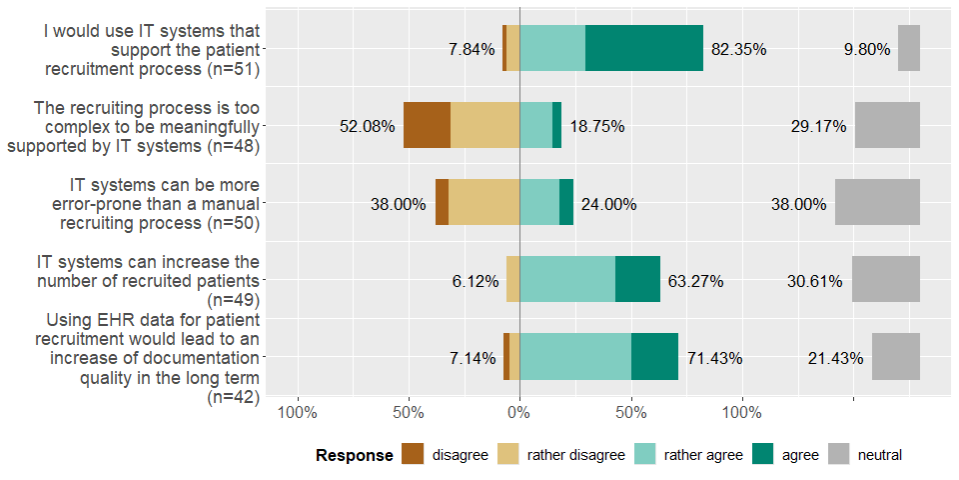
Our participants' expectations of a patient recruitment system. EHR: electronic health record, IT: information technology.

As part of the web-based survey, we were interested in our participants' expectations concerning the capabilities of a PRS. Figure 4 illustrates the participants' selection of predefined multiple-choice answers in percentages, split in accordance with the 5-item scale. In general, our participants would want to use such a tool, and they assume that this type of system could be capable of supporting complex recruitment processes. They also expect that this tool would increase the number of recruited patients as well as the documentation quality, while potentially lowering errors.

### Requirements for Patient Recruitment Systems

In the following section, we relate our findings to those reported in the literature and discuss functional and technical requirements toward future PRS.

#### Functional Requirements

We explicitly asked our interview participants to name their expectations and requirements toward a PRS. Below, we summarize the most relevant ones.

#### Overview of Patients

Almost all of our participants wanted an overview of potentially eligible patients for their study. Namely, one should be able to mark participants, make notes, and track the recruitment status. In addition, one should be able to manually add or remove patients from the recruitment list. Some participants specifically requested that patient summaries be integrated into existing systems.

#### Overview of Trials

Participants expressed that the PRS should include a module to manage studies and inclusion criteria, and that a link to the ClinicalTrials.gov study registry would also be useful. Many participants would also like to see a registry of all ongoing studies at the clinic. In some cases, cross-site recruitment support with other hospitals was desired.

#### Notifications

Furthermore, our participants wanted to instantly be notified when an eligible patient was found. Those notifications should also be manageable by the user to fit individual preferences as well as the workflow and roles [12,14].

#### Search

Additionally, our participants mentioned that a PRS absolutely needs to offer sophisticated search options; for example, for feasibility tests.

#### User Management and Interface

Our participants mentioned that the PRS must contain a sophisticated rights concept to account for the various roles in the study and at the clinical center, while the PRS must be easy to use by means of a clear and fast user interface.

#### Learn from Workarounds

Our interviewees mentioned that certain programs—for instance, Microsoft Office tools—are currently being used for patient recruitment, although these were not designed for this purpose. Future studies should therefore investigate how and for what these programs are used, so as to extract further functional requirements for the PRS.

#### Integration Into Workflows

Integrating PRS into clinical workflows can change various staff tasks and roles. Currently, many processes are carried out manually and are paper-based. For example, research staff might sift through recruitment proposals instead of manually screening all patient records as they do now. Physicians would no longer have to search for patients during rounds. However, we still assume that they would still be busy with other recruitment tasks, such as further diagnostics or patient education. However, according to Good Clinical Practice, the final screening task should always be performed by a physician. A PRS could only make recruiting recommendations. Before a PRS is ready for real-world use, further research should examine its usability, effectiveness, and workflow integration or modification. Another round of development may be required to adapt the PRS to the new workflows and to ensure its acceptance and effectiveness.

#### Integration Into Technical Infrastructures

Schreiweis et al [16] suggest that a PRS needs to be integrated with existing systems, especially to avoid additional documentation burden on the staff. According to Campbell et al [4], a lack of integration of systems is one of the reasons why many studies do not achieve the required recruitment numbers. We argue that a PRS must be integrated or at least connected to the existing technical infrastructure to make data accessible to the PRS in a timely manner. This would also have the advantage of reusing central user and rights management and would avoid further tool changes. Often, data required for recruitment are distributed across multiple systems. Therefore, a PRS must either be able to handle disparate data sources or the facility establish and advance data integration protocol to create a unified, hospital-wide research repository. Using data from standardized research repositories (such as i2b2 or the Observational Medical Outcomes Partnership Common Data Model) has the major advantage that the PRS can be reused at any site implementing such a repository.

#### Data Availability and Accessibility

Our participants identified various criteria and data types that are needed to search and screen for eligible patients. They also mentioned that sophisticated searches in digital documents and data repositories are rarely possible. This inaccessibility may have various reasons, including the following:

1. Data are not collected, meaning that the data needed to compare a patient with trial criteria are not consistently collected for every patient and thus not available at all,
2. Data are analog (paper-based) or a digital version is not available and thus not accessible to the systems,
3. Data are unstructured, as in medical letters and thus not easily processed and searched,
4. Data quality is insufficient; for example, incomplete data or with documentation errors, and is thus not reliable,
5. Interoperability or rights are limited, meaning that data are present but in an inaccessible system,
6. Users are not provided with the right tools; for example, because there simply is no system that allows for a sophisticated search or the system is too complex for the users.

Each of these reasons points to specific criteria that are needed for the success of future PRS: relevant data must be collected, digitized, structured, quality-checked, and made available in a system that respects data privacy regulations and that is not too complex for the user. This is in line the findings of Trinczek et al [18]. Doods et al [27] developed a comprehensive clinical trial data inventory. They reviewed which data types were available in 9 European hospitals. Their results clearly show that hospitals are far from having complete data available for PRS. Nevertheless, their generated data lists can serve as an agenda for what data needs to be addressed and with what priority.

## Discussion

### Principal Findings

Low recruitment is one of the major reasons why clinical trials fail. Many studies indicate that patient recruitment systems can increase recruitment effectiveness and efficiency. To ensure that PRSs are successfully integrated in clinical environments in the long term, an in-depth analysis of the system context and requirements is needed [12,14,16]. Our study aims at identifying many aspects needed for a successful PRS and confirms many findings of related works, but also extends them in various ways. Similar to Becker et al [28], we found that approximately half of our participants do not use any software, and that most of those who do adopt a system (eg, Microsoft Excel), adopt one that is not intended for patient recruitment. Successful recruitment highly depends on the staff, particularly their motivation and knowledge [12,28,29] and interpersonal communication. A PRS, which can identify and screen patients, could change particular duties of staff members and possibly affect their workflows and collaboration. A future PRS will also need to be as flexible as the recruitment workflows, especially regarding when and how it is used. Similar to the study of Trinczek et al [18], our results show that a tremendous amount of work is done manually and is paper-based. Our results also confirm that highly specific searches in the clinical data repositories are not possible or very limited. Instead, our participants rely on various, often paper-based sources, such as consultation schedules and medical reports, to find and screen eligible patients. Doods et al [27] reported that only a fragment of the data needed for clinical trial feasibility studies is readily available and accessible in European hospitals. Furthermore, we show which data our participants regard as most important for patient recruitment as well as from which sources they get those data. It should be noted, however, that according to Gulden et al [30] not all data elements can be meaningfully queried by IT systems; for example, pregnancy status or capacity to consent is rarely documented.

We also demonstrated which concrete requirements a PRS needs to fulfill to be successful. Overall, our results confirm and extend the list of requirements reported by Schreiweis et al [16]. In addition, we have discussed various functional and technical requirements and provided concrete recommendations for the design, development, and integration of future PRS. Prospective users should be involved in the design and development process to ensure that the system meets their needs and capabilities. Sophisticated user studies should furthermore assess the quality of the systems well as their effectiveness for patient recruitment.

### Limitations and Methodological Implications

In total, there are 33 university hospitals in Germany. In this study, we recruited 56 participants from 10 sites, which indicates that our sample is not necessarily representative of the status quo in Germany. We could neither include all hospitals nor recruit participants from each ward, department, and clinic from the hospitals involved in this study. Furthermore, the numbers of participants in the interviews and web-based questionnaires were not evenly distributed across the sites. The web-based survey was answered by approximately 2-3 persons per site. Few sites were able to recruit additional participants, which adds more weightage to the responses of those sites. Since procedures can vary between wards as they vary between hospitals, we do not consider this problematic. On the contrary, to obtain highly representative findings, it was important to us to recruit a large number of clinicians from as many different specialties as possible. Further, we did not enforce the semistructured interviews to be conducted in person, causing some participants to opt for answering the questions in written form. This resulted in short or missing responses in some cases. We did not exclude those participants from our analysis as they all presented valuable insights. As some of those insights were only mentioned by a single participant, an exclusion of incomplete responses would mean to a loss of valuable findings. In a qualitative analysis, obtaining the same number of codes for every participant and every question can generally not be assured, which implies that even if all responses were complete, they might not contain more findings. Thus, we were able to obtain insights and requirements from a larger group of participants and specialist areas.

### Conclusions and Future Prospects

Problems in patient recruitment are common in clinical trials. There are various ambitions to overcome this issue by means of a patient recruitment system, which supports the identification of potential participants. However, those attempts are not based on a profound investigation of the status quo of recruitment, the workflows and environment in which a PRS would have to be embedded, which risks user acceptance and therefore the success of such a system. We present detailed findings on the recruitment workflows, tasks, and timing. Furthermore, we report on the momentary IT support and discuss functional and technical requirements for patient recruitment systems. We showed that identifying eligible patients is still associated with significant manual effort. To enable the use of a PRS, data from disparate sources will need to be made available. Lastly, we contribute and discuss concrete technical challenges for patient recruitment systems, including requirements for features, data, infrastructure, and workflow integration. Regarding the next step, we suggest that our findings should be translated into interface and interaction concepts, which may then serve as a basis for development. We argue that users need to be involved in both steps, concept design and system testing, to ensure the success of the PRS.

## Abbreviations

EHR: electronic health record
HIS: hospital information system
IT: information technology
MIRACUM: Medical Informatics in Research and Care in University Medicine
PRS: patient recruitment system
